# Supplementary material for: Modeling glioblastoma heterogeneity as a dynamic network of cell states
Source: Mol Syst Biol. 2021 Sep 16;17(9):e10105. doi: 10.15252/msb.202010105 (PMC8444284; doi:10.15252/msb.202010105)
Supplement: Supplementary file 6 — Source Data for Figure 5 [file MSB-17-e10105-s004.zip › Figure5A_sourcedata/GSEA_3071/nosigdb_stateV.GseaPreranked.1622795159954/NB_KI_CLUSTER17.html]

Details for gene set NB\_KI\_CLUSTER17[GSEA]

|  || Dataset | state43071 |
| Phenotype | NoPhenotypeAvailable |
| Upregulated in class | na\_neg |
| GeneSet | NB\_KI\_CLUSTER17 |
| Enrichment Score (ES) | -0.4280663 |
| Normalized Enrichment Score (NES) | -2.394481 |
| Nominal p-value | 0.0 |
| FDR q-value | 4.3243246E-5 |
| FWER p-Value | 0.001 |
Table: GSEA Results Summary

  

Fig 1: Enrichment plot: NB\_KI\_CLUSTER17      
 Profile of the Running ES Score & Positions of GeneSet Members on the Rank Ordered List

  

| PROBE | GENE SYMBOL | GENE\_TITLE | RANK IN GENE LIST | RANK METRIC SCORE | RUNNING ES | CORE ENRICHMENT || 1 | NFIA |  |  | 42 | 1.604 | 0.0125 | No |
| 2 | EMP2 |  |  | 59 | 1.511 | 0.0288 | No |
| 3 | GPM6B |  |  | 121 | 1.326 | 0.0340 | No |
| 4 | NFIC |  |  | 172 | 1.227 | 0.0401 | No |
| 5 | IL1RAP |  |  | 183 | 1.204 | 0.0537 | No |
| 6 | DAG1 |  |  | 267 | 1.095 | 0.0517 | No |
| 7 | PLPP3 |  |  | 323 | 1.042 | 0.0545 | No |
| 8 | SPRY1 |  |  | 389 | 0.959 | 0.0543 | No |
| 9 | HEG1 |  |  | 592 | 0.805 | 0.0257 | No |
| 10 | PLAT |  |  | 651 | 0.773 | 0.0245 | No |
| 11 | PMEPA1 |  |  | 718 | 0.740 | 0.0212 | No |
| 12 | FGFR1 |  |  | 862 | 0.673 | 0.0023 | No |
| 13 | EDNRB |  |  | 911 | 0.654 | 0.0015 | No |
| 14 | NBL1 |  |  | 973 | 0.630 | -0.0022 | No |
| 15 | PLXND1 |  |  | 978 | 0.628 | 0.0051 | No |
| 16 | TGFBR3 |  |  | 1203 | 0.560 | -0.0309 | No |
| 17 | GNG11 |  |  | 1219 | 0.556 | -0.0266 | No |
| 18 | ITM2B |  |  | 1244 | 0.547 | -0.0242 | No |
| 19 | TSPAN15 |  |  | 1287 | 0.535 | -0.0254 | No |
| 20 | LTBP4 |  |  | 1436 | 0.496 | -0.0476 | No |
| 21 | KCTD12 |  |  | 1494 | 0.481 | -0.0524 | No |
| 22 | ERBB3 |  |  | 1521 | 0.477 | -0.0513 | No |
| 23 | EGR1 |  |  | 1691 | 0.438 | -0.0782 | No |
| 24 | GOLIM4 |  |  | 1754 | 0.427 | -0.0847 | No |
| 25 | WWTR1 |  |  | 1794 | 0.419 | -0.0868 | No |
| 26 | PSAP |  |  | 1855 | 0.410 | -0.0931 | No |
| 27 | ZEB2 |  |  | 2009 | 0.386 | -0.1176 | No |
| 28 | SPRED1 |  |  | 2166 | 0.366 | -0.1430 | No |
| 29 | NDRG2 |  |  | 2325 | 0.345 | -0.1690 | No |
| 30 | TCIM |  |  | 2438 | 0.330 | -0.1863 | No |
| 31 | FNDC3B |  |  | 2617 | 0.308 | -0.2166 | No |
| 32 | SH3PXD2A |  |  | 2831 | 0.286 | -0.2540 | No |
| 33 | CLEC11A |  |  | 2957 | 0.273 | -0.2746 | No |
| 34 | CALU |  |  | 3001 | 0.269 | -0.2794 | No |
| 35 | IL6ST |  |  | 3233 | -0.257 | -0.3206 | No |
| 36 | TNFAIP2 |  |  | 3314 | -0.269 | -0.3326 | No |
| 37 | TNS2 |  |  | 3520 | -0.303 | -0.3682 | No |
| 38 | CNN3 |  |  | 3751 | -0.339 | -0.4081 | No |
| 39 | SVIL |  |  | 3763 | -0.342 | -0.4058 | No |
| 40 | CTSO |  |  | 3774 | -0.344 | -0.4033 | No |
| 41 | KLF4 |  |  | 3866 | -0.361 | -0.4162 | No |
| 42 | TUBB6 |  |  | 3904 | -0.368 | -0.4186 | No |
| 43 | EPB41L2 |  |  | 3911 | -0.369 | -0.4151 | No |
| 44 | TSPAN4 |  |  | 3916 | -0.369 | -0.4111 | No |
| 45 | RPS27L |  |  | 3927 | -0.373 | -0.4082 | No |
| 46 | TRAM1 |  |  | 3935 | -0.375 | -0.4048 | No |
| 47 | CD59 |  |  | 4024 | -0.394 | -0.4167 | No |
| 48 | CLIC4 |  |  | 4075 | -0.404 | -0.4211 | No |
| 49 | UGCG |  |  | 4112 | -0.412 | -0.4228 | Yes |
| 50 | JUN |  |  | 4135 | -0.418 | -0.4217 | Yes |
| 51 | SPATS2L |  |  | 4148 | -0.422 | -0.4186 | Yes |
| 52 | CREB3L2 |  |  | 4178 | -0.430 | -0.4186 | Yes |
| 53 | PDLIM2 |  |  | 4180 | -0.432 | -0.4133 | Yes |
| 54 | ITGAV |  |  | 4207 | -0.442 | -0.4126 | Yes |
| 55 | PHACTR2 |  |  | 4213 | -0.444 | -0.4079 | Yes |
| 56 | APOE |  |  | 4229 | -0.449 | -0.4050 | Yes |
| 57 | NECTIN2 |  |  | 4260 | -0.458 | -0.4049 | Yes |
| 58 | RCN1 |  |  | 4276 | -0.467 | -0.4018 | Yes |
| 59 | ARHGAP24 |  |  | 4315 | -0.476 | -0.4030 | Yes |
| 60 | NOTCH2 |  |  | 4355 | -0.488 | -0.4043 | Yes |
| 61 | BLVRB |  |  | 4371 | -0.491 | -0.4009 | Yes |
| 62 | HNMT |  |  | 4379 | -0.494 | -0.3959 | Yes |
| 63 | RASSF4 |  |  | 4410 | -0.504 | -0.3952 | Yes |
| 64 | MT1X |  |  | 4481 | -0.530 | -0.4019 | Yes |
| 65 | CCDC80 |  |  | 4510 | -0.546 | -0.4003 | Yes |
| 66 | FTH1 |  |  | 4515 | -0.547 | -0.3940 | Yes |
| 67 | WIPI1 |  |  | 4599 | -0.589 | -0.4024 | Yes |
| 68 | SPON2 |  |  | 4603 | -0.591 | -0.3954 | Yes |
| 69 | ANXA5 |  |  | 4616 | -0.601 | -0.3900 | Yes |
| 70 | LOXL2 |  |  | 4624 | -0.605 | -0.3836 | Yes |
| 71 | TAX1BP3 |  |  | 4631 | -0.609 | -0.3770 | Yes |
| 72 | MYDGF |  |  | 4704 | -0.646 | -0.3826 | Yes |
| 73 | RAB13 |  |  | 4786 | -0.701 | -0.3892 | Yes |
| 74 | S100A13 |  |  | 4791 | -0.705 | -0.3809 | Yes |
| 75 | IGFBP6 |  |  | 4800 | -0.715 | -0.3733 | Yes |
| 76 | RHOB |  |  | 4820 | -0.731 | -0.3676 | Yes |
| 77 | DNAJB4 |  |  | 4833 | -0.739 | -0.3604 | Yes |
| 78 | NPC2 |  |  | 4841 | -0.750 | -0.3521 | Yes |
| 79 | LRP10 |  |  | 4843 | -0.751 | -0.3427 | Yes |
| 80 | RHOC |  |  | 4868 | -0.776 | -0.3373 | Yes |
| 81 | CSTB |  |  | 4901 | -0.811 | -0.3331 | Yes |
| 82 | ITIH5 |  |  | 4934 | -0.850 | -0.3284 | Yes |
| 83 | ITPRIPL2 |  |  | 4943 | -0.860 | -0.3189 | Yes |
| 84 | TNS3 |  |  | 4973 | -0.890 | -0.3131 | Yes |
| 85 | CTSB |  |  | 5007 | -0.948 | -0.3073 | Yes |
| 86 | EMP1 |  |  | 5023 | -0.979 | -0.2976 | Yes |
| 87 | GBP1 |  |  | 5051 | -1.031 | -0.2896 | Yes |
| 88 | REXO2 |  |  | 5053 | -1.035 | -0.2765 | Yes |
| 89 | ANXA2 |  |  | 5055 | -1.036 | -0.2634 | Yes |
| 90 | PLA2G16 |  |  | 5067 | -1.067 | -0.2518 | Yes |
| 91 | FTL |  |  | 5072 | -1.077 | -0.2388 | Yes |
| 92 | PLXDC2 |  |  | 5096 | -1.138 | -0.2286 | Yes |
| 93 | PROCR |  |  | 5097 | -1.139 | -0.2140 | Yes |
| 94 | ANXA4 |  |  | 5106 | -1.172 | -0.2005 | Yes |
| 95 | PHLDA3 |  |  | 5150 | -1.320 | -0.1918 | Yes |
| 96 | MT1E |  |  | 5154 | -1.323 | -0.1755 | Yes |
| 97 | GPX8 |  |  | 5171 | -1.418 | -0.1603 | Yes |
| 98 | MATN2 |  |  | 5200 | -1.557 | -0.1458 | Yes |
| 99 | LRP1 |  |  | 5234 | -1.801 | -0.1290 | Yes |
| 100 | PCOLCE |  |  | 5248 | -1.999 | -0.1059 | Yes |
| 101 | CFH |  |  | 5257 | -2.141 | -0.0800 | Yes |
| 102 | DAB2 |  |  | 5261 | -2.217 | -0.0521 | Yes |
| 103 | FBN1 |  |  | 5263 | -2.234 | -0.0236 | Yes |
| 104 | AHNAK |  |  | 5268 | -2.309 | 0.0052 | Yes |
Table: GSEA details [plain text format]

  

Fig 2: NB\_KI\_CLUSTER17: Random ES distribution      
 Gene set null distribution of ES for **NB\_KI\_CLUSTER17**
